# Impact of clonal plasma cells in autografts on outcomes in high-risk multiple myeloma patients

**DOI:** 10.1038/s41408-023-00842-6

**Published:** 2023-05-03

**Authors:** Oren Pasvolsky, Denái R. Milton, Mikael Rauf, Sassine Ghanem, Adeel Masood, Ali H. Mohamedi, Mark R. Tanner, Qaiser Bashir, Samer Srour, Neeraj Saini, Paul Lin, Jeremy Ramdial, Yago Nieto, Guilin Tang, Hans C. Lee, Krina K. Patel, Partow Kebriaei, Sheeba K. Thomas, Donna M. Weber, Robert Z. Orlowski, Katy Rezvani, Richard Champlin, Elizabeth J. Shpall, Pei Lin, Muzaffar H. Qazilbash

**Affiliations:** 1 Department of Stem Cell Transplantation and Cellular Therapy, The University of Texas M.D. Anderson Cancer Center, Houston, TX USA; 2 Institute of Hematology, Davidoff Cancer Center, Rabin Medical Center, Petah-Tikva, Israel; 3 Sackler Faculty of Medicine, Tel Aviv University, Tel Aviv, Israel; 4 Department of Biostatistics, The University of Texas M.D. Anderson Cancer Center, Houston, TX USA; 5 Department of Medicine, Alpert Medical School of Brown University, Providence, RI USA; 6 Department of Hematopathology, The University of Texas M.D. Anderson Cancer Center, Houston, TX USA; 7 Department of Lymphoma/Myeloma, The University of Texas M.D. Anderson Cancer Center, Houston, TX USA

**Keywords:** Myeloma, Prognosis

## Abstract

Most patients with multiple myeloma (MM) undergoing autologous hematopoietic stem cell transplantation (autoHCT) eventually relapse, perhaps due to the presence of clonal plasma cells (CPC) in the autograft. We conducted a retrospective analysis to evaluate the impact of CPC in the autograft on the outcomes of high-risk chromosomal abnormalities (HRMM) patients undergoing autoHCT between 2008 and 2018. Patients were divided into CPC+ or CPC− in the autograft by next-generation flow cytometry (NGF). There were 75 CPC + autografts (18%) and 341 CPC− (82%). The CPC + group was less likely to achieve MRD-negative complete remission post-transplant (11% vs. 42%; *p* < 0.001). Median progression free survival (PFS) and overall survival (OS) were (12.8 vs. 32.1 months) and (36.4 vs. 81.2 months) in the CPC + and CPC− groups, respectively (both *p* < 0.001). Also in the subset of patients with MRD-negative ≥VGPR prior to autoHCT, those with CPC + autografts had inferior PFS (HR 4.21, *p* = 0.006) and OS (HR 7.04, *p* = 0.002) compared to CPC-. In multivariable analysis, the degree of CPC positivity in the autograft was independently predictive of worse PFS (HR 1.50, *p* = 0.001) and OS (HR 1.37, *p* = 0.001). In conclusion, both the presence and degree of CPC in the autograft were highly predictive of inferior PFS and OS.

## Introduction

High dose chemotherapy and autologous hematopoietic stem cell transplantation (autoHCT) is considered standard of care as part of first line therapy for patients with multiple myeloma (MM) [1]. However, patients with high-risk cytogenetic features (high risk MM, HRMM) have inferior outcomes after autoHCT compared to patients with standard risk disease. A Center for International Blood and Marrow Transplant Research (CIBMTR) analysis showed a 3 year post-transplant progression free survival (PFS) rate of 37% vs. 49%, and overall survival (OS) rate of 72% vs. 85%, in patients with HRMM compared to those with standard risk MM, respectively [2].

The significance of aberrant clonal plasma cells (CPC) in the autografts collected for autoHCT remains a subject of debate. In a study of 76 patients reported by Vogel et al., those with CPC graft contamination of >4.5 × 10^5^ plasma cells/kg body weight by flow cytometry had a high risk of early disease progression [3]. A follow-up report found that patients with a high degree of CPC autograft contamination had inferior OS compared to those with a low degree of contamination (median OS: 53 vs. 114 months, respectively) [4]. Several small studies provided conflicting results regarding the impact of CPC in the autograft on PFS in patients with MM [5–7]. More recently, a study involving 199 patients showed that autograft contamination by MM cells was associated with a higher risk of delaying or not achieving complete remission (CR) and minimal residual disease (MRD)-negativity post-transplant [8]. There was no difference in PFS or OS between the two groups, though the follow-up was relatively short.

In the current study, we conducted a single center retrospective analysis to evaluate the impact of autograft contamination by MM cells in patients with HRMM undergoing autoHCT.

## Methods

### Study design and participants

We searched our institution’s database for adult patients with HRMM who underwent autoHCT between 2008 and 2018. Evaluation for CPC by 6-color next-generation flow cytometry (NGF) was performed on the collected apheresis products, with a sensitivity of 0.001–0.003% depending on sample quality. Of note, 4-color flow cytometry was used before 2012, with a sensitivity of 0.05%. The patients were divided into two groups: CPC + and CPC− in the autograft by NGF. Since not all collected autograft products are infused at autoHCT, and some bags may be CPC + or CPC− for the same patient, we also evaluated the impact of infusion of CPC + autograft products on patient outcomes. MRD status in bone marrow samples was assessed using 8-color NGF. The sensitivity of our assay is 1/10^−5^ cells (0.001%) based on acquisition and analysis of at least 2 million events.

The University of Texas MD Anderson Cancer Center Institutional Review Board approved this retrospective study. The research was conducted in accordance with the Declaration of Helsinki and the 1996 Health Insurance Portability and Accountability Act guidelines.

### FISH analysis

Fluorescence in situ hybridization (FISH) was used to identify high-risk cytogenetic abnormalities of del17p/TP53 deletion, t(4;14)/IGH::FGFR3, t(14;16)/IGH::MAF, and 1q21/CKS1B gain (3 copies of CKS1B) or amplification (≥4 copies of CKS1B). The cut-off values established in our clinical cytogenetics laboratory were 0.4% for IGH/FGFR3 or IGH::MAF rearrangement; 4.7% for TP53 deletion and 4.2% for monosomy 17.

### Statistical methods

We compared the day 100 post autoHCT response, the best response, PFS and OS between CPC+ and CPC− autograft groups. Response between CPC+ and CPC− autograft groups was evaluated using Fisher’s exact test, while response by degree of autograft CPC+ bag infused was assessed by Wilcoxon rank-sum test. PFS time was computed from autoHCT date to date of disease progression or death (if died without disease progression) or the last follow-up date. Patients who were alive and did not experience progression of disease at the last follow-up date were censored. OS time was computed from date of autoHCT to last known vital sign, and patients alive at the last follow-up date were censored. OS and PFS were estimated using the Kaplan–Meier method, and differences between groups were assessed using the log-rank test. Associations between outcomes and variables of interest were determined using univariate and multivariable Cox proportional hazards regression models.

Statistical analyses were performed using SAS 9.4 for Windows (by SAS Institute Inc., Cary, NC). All statistical tests used a significance (alpha) level of 5%. No adjustments for multiple testing were made.

#### Data sharing statement

The data that support the findings of this study are available on request from the corresponding author.

## Results

### Patients and disease characteristics

416 HRMM patients were included in the study, with a median age of 62.4 years (range 31.7–83.0), and 57% were male. Seventy-five patients (18%) had CPC + status while 341 (82%) were CPC−. A lower percentage of patients in the CPC + group received the bortezomib, lenalidomide, and dexamethasone (VRD) induction regimen prior to transplant compared to the CPC− group (24% vs. 42%, *p* = 0.004), yet there was no difference in the duration of induction between the two groups (*p* = 0.37) A lower percentage of patients in the CPC + group achieved ≥VGPR and MRD negative status after induction compared to the CPC− group (32% vs. 62%; *p* < 0.001 and 8% vs. 40%; *p* < 0.001, respectively). Del[17p] and 1q+ cytogenetic abnormalities were significantly more prevalent in patients with CPC + autografts compared to those with CPC− autografts (45% vs. 28%, *p* = 0.007 and 68% vs. 45%, *p* < 0.001). There was no significant difference in conditioning regimens used between the two groups (*p* = 0.73). Patient characteristics according to autograft CPC status are summarized in Table 1.

**Table 1:** Patient characteristics.

Measure	Autograft CPC			
	All	Positive	Negative	*p* value^a^
	(*N* = 416)	(*N* = 75)	(*N* = 341)	
Gender, *n* (%)				0.70
Male	236 (57)	41 (55)	195 (57)	
Female	180 (43)	34 (45)	146 (43)	
Age at autoHCT (years)				0.032^b^
Median (Range)	62.4 (31.7–83.0)	63.8 (43.5–83.0)	62.1 (31.7–79.9)	
Year of autoHCT, *n* (%)				0.013
2008–2011	55 (13)	17 (23)	38 (11)	
2012–2018	361 (87)	58 (77)	303 (89)	
Induction treatment, *n* (%)				
VRD	159 (40)	18 (24)	141 (42)	0.004
VCD	64 (16)	14 (19)	50 (15)	0.38
KRD	64 (16)	11 (15)	53 (16)	1.00
VD	71 (17)	16 (21)	55 (16)	0.31
VTD	6 (1)	4 (5)	2 (1)	0.011
Chemotherapy	16 (4)	2 (3)	14 (4)	0.75
RD	15 (4)	7 (9)	8 (2)	0.009
Other	18 (4)	3 (4)	15 (4)	1.00
Duration of induction, Median (days)	107.0 (19–511)	106.5 (22–511)	111.5 (19–495)	0.37^b^
Mobilization type, *n* (%)				0.39
No chemotherapy	348 (84)	60 (80)	288 (84)	
Chemotherapy	68 (16)	15 (20)	53 (16)	
ISS, *n* (%)				0.48
I	118 (34)	17 (27)	101 (35)	
II	122 (35)	24 (39)	98 (34)	
III	107 (31)	21 (34)	86 (30)	
Unknown	69	13	56	
RISS, *n* (%)				0.19
I	49 (16)	5 (9)	44 (17)	
II	203 (65)	35 (65)	168 (65)	
III	59 (19)	14 (26)	45 (18)	
Unknown	105	21	84	
KPS, *n* (%)				0.24
<90	165 (42)	35 (49)	130 (41)	
≥90	227 (58)	37 (51)	190 (59)	
Unknown	24	3	21	
HCT-CI, *n* (%)				0.45
≤3	321 (77)	55 (73)	266 (78)	
>3	95 (23)	20 (27)	75 (22)	
Conditioning regimen, *n* (%)				0.73
Bu/Mel based	71 (17)	14 (19)	57 (17)	
Mel	345 (83)	61 (81)	284 (83)	
Response prior to autoHCT, *n* (%)				<0.001
sCR/CR	55 (13)	1 (1)	54 (16)	
nCR/VGPR	179 (43)	23 (31)	156 (46)	
PR	135 (32)	31 (41)	104 (30)	
SD	13 (3)	3 (4)	10 (3)	
PD	17 (23)	17 (5)		
MRD prior to autoHCT, *n* (%)				<0.001
Negative	134 (34)	6 (8)	128 (40)	
Positive	261 (66)	66 (92)	195 (60)	
Unknown	21	3	18	
del 17p, *n* (%)				0.007
Negative	259 (69)	35 (55)	224 (72)	
Positive	115 (31)	29 (45)	86 (28)	
NA	42	11	31	
t(4;14), *n* (%)				0.71
Negative	114 (59)	22 (63)	92 (58)	
Positive	79 (41)	13 (37)	66 (42)	
NA	223	40	183	
t(14;16), *n* (%)				0.24
Negative	97 (78)	18 (90)	79 (76)	
Positive	27 (22)	2 (10)	25 (24)	
NA	292	55	237	
Any 1q+, *n* (%)				<0.001
Negative	203 (49)	51 (68)	152 (45)	
Positive	213 (51)	24 (32)	189 (55)	
Maintenance therapy, *n* (%)				0.24
Len alone/RD	176 (42)	25 (33)	151 (44)	
PI alone	50 (12)	7 (9)	43 (13)	
Len+PI	41 (10)	9 (12)	32 (9)	
Len+Elo	29 (7)	5 (7)	24 (7)	
IMid alone	3 (1)	1 (1)	2 (1)	
None	117 (28)	28 (37)	89 (26)	

*CPC* clonal plasma cells, *n* number, *autoHCT* autologous hematopoietic stem cell transplant, *VRD* bortezomib, lenalidomide, dexamethasone, *VCD* bortezomib, cyclophosphamide, dexamethasone, *KRD* carfilzomib, lenalidomide, dexamethasone, *VD* bortezomib, dexamethasone, *VTD* bortezomib, thalidomide, dexamethasone, *RD* lenalidomide, dexamethasone, *KPS* karnofsky performance status, *sCR* stringent complete response, *CR* complete response, *VGPR* very good partial response, *PR* partial response, *SD* stable disease, *PD* progressive disease, *MRD* minimal residual disease, *NA* not available, *Len* lenalidomide, *PI* proteasome inhibitor, *ELO* elotuzumab.

^a^Fisher’s exact test or generalization.

^b^Wilcoxon rank-sum test.

### Outcomes

Median follow-up for the whole cohort was 35.7 (range 0.3–139.5) months. 100-day and best post-autoHCT CR rates in the CPC+ and CPC− groups were 8% vs. 33% (*p* < 0.001), and 19% vs. 54% (*p* < 0.001), respectively. Patients in the CPC + group were less likely to have MRD-negative CR post-transplant (11% vs. 42%; *p* < 0.001). Median PFS in the CPC + vs. CPC− group was 12.8 vs. 32.1 months (*p* < 0.001), and median OS was 36.4 vs. 81.2 months (*p* < 0.001).

**Fig. 1:**
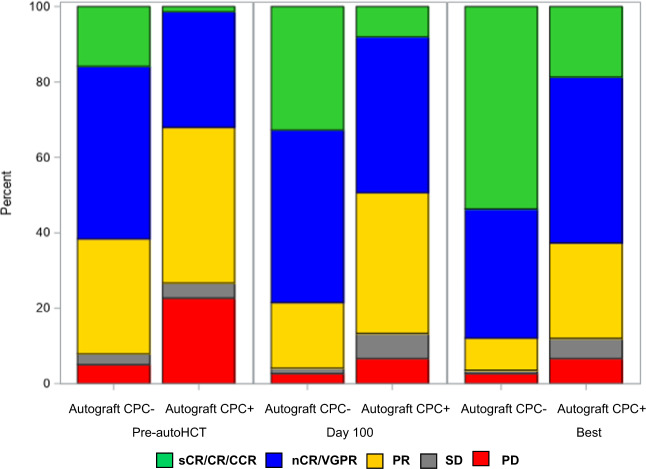
Hematological responses according to the presence of CPC in the autograft. Disease responses for the CPC+ and CPC− groups prior to transplant, at day 100 post-transplant and at best response post-transplant.

The degree of autograft CPC involvement was inversely associated with post-transplant day 100 response: the maximal autograft CPC level was 0.02% (standard deviation [SD] 0.02) for patients who achieved ≥VGPR vs. 1.04% (SD 2.59) for those who achieved ≤PR at day 100 post-transplant (*p* = 0.003). Similarly, the maximal autograft CPC level was inversely associated with best post-transplant response: mean maximal autograft CPC level was 0.06% (SD 0.16) for patients who achieved ≥VGPR vs. vs. 1.55% (SD 3.17) for those who achieved ≤PR (*p* = 0.004). Pre- and post-transplant responses for the CPC+ and CPC− groups are depicted in Fig. 1.

**Fig. 2:**
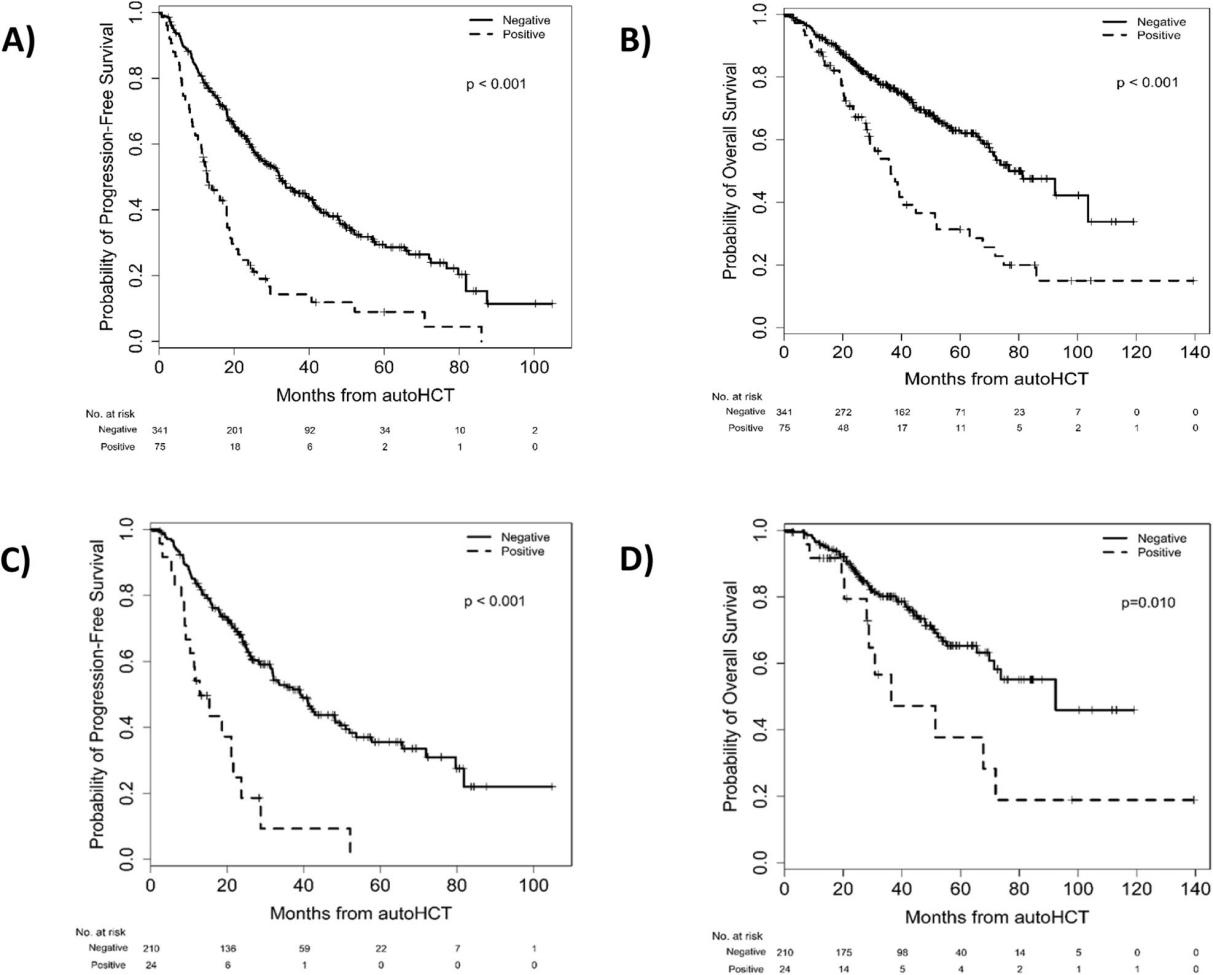
Progression free and overall survival according to the presence of CPC in the autograft. **A** PFS in the entire cohort. **B** OS in the entire cohort. **C** PFS in the subgroup of patients with ≥VGPR prior to transplant. **D** OS in the subgroup of patients with ≥VGPR prior to transplant.

When assessing the whole cohort of patients, those with CPC + autografts had significantly worse PFS (Fig. 2A, *p* < 0.001) and OS (Fig. 2B, *p* < 0.001) compared to those with CPC- autografts. Of the patients who achieved ≥VGPR prior to autoHCT, those with CPC + autografts had inferior PFS (hazard ratio (HR) [95% CI]: 3.38 [2.05–5.58], *p* < 0.001; Fig. 2C) and OS (HR 2.29 [1.20–4.40], *p* = 0.013; Fig. 2D) compared to the CPC− group. Also in patients who achieved MRD-negative ≥VGPR prior to autoHCT, those with CPC + autografts had inferior PFS (HR 4.21 [1.50–11.81], *p* = 0.006) and OS (HR 7.04 [2.05–24.20], *p* = 0.002) compared to those of the CPC− group. In a multivariable analysis (MVA), the degree of CPC positivity in the autograft was independently predictive of worse PFS (HR 1.50 [1.17–1.90]; *p* = 0.001) and OS (HR 1.37 [1.13–1.67]; *p* = 0.001). MVA showed that infusion of CPC + autograft products was predictive of worse PFS (HR 2.72 [1.82–4.08]; *p* < 0.001) but not significantly worse OS (HR 1.47 [0.86–2.49]; *p* = 0.16). ISS stage II and III disease were associated with worse PFS compared to ISS stage I in MVA [(HR 1.47 [1.05–2.06]; *p* = 0.026) and (HR 1.55 [1.09–2.21]; *p* = 0.014), respectively]. HCT-CI score >3 was associated with worse OS (HR 1.50 [1.01–2.22]; *p* = 0.044), whereas post-transplant maintenance was associated with a trend toward improved OS (HR 0.68 [0.46–1.00]; *p* = 0.05) in MVA. MVA for PFS and OS are shown in Table 2 and Table 3, respectively. Univariate analyses for PFS and OS are shown in Supplemental Table 1 and Supplemental Table 2, respectively. Within the CPC− and CPC + groups, hematological responses prior to autoHCT and at day 100 after autoHCT mostly retained their statistical significance for both PFS and OS (Supplementary Figs. 1–5 and 7–8). However, within the CPC + group, hematological responses prior to autoHCT did not predict OS (*p* = 0.60, Supplementary Fig. 6).

**Table 2:** Summary of multivariable assessment for progression free survival (PFS).

Parameter	Hazard ratio (95% CI)	*p* value
Autograft CPC status		
Positive vs. Negative	2.03 (1.48, 2.80)	<0.001
ISS		
II vs. I	1.47 (1.05, 2.06)	0.026
III vs. I	1.55 (1.09, 2.21)	0.014
KPS		
≥90 vs. <90	0.95 (0.73, 1.24)	0.72
HCT-CI		
>3 vs. ≤3	1.19 (0.88, 1.62)	0.25
Prior response		
PR or worse vs. VGPR or better	1.24 (0.93, 1.64)	0.14
Prior MRD response		
Positive vs. Negative	1.61 (1.17, 2.22)	0.004
Maintenance therapy^a^		
Yes vs. No	1.20 (0.86, 1.66)	0.28
Positive bag infused^b^		
Yes vs. No	2.72 (1.82, 4.08)	<0.001
Degree of autograft MRD positivity – average^b^	1.50 (1.17, 1.90)	0.001

*CI* Confidence interval, *MRD* Minimal residual disease, *ISS* international staging system, *KPS* Karnofsky performance status, *HCT-CI* Hematopoietic cell transplant comorbidity index.

^a^Included in the model as a time-dependent covariate.

^b^Replaces autograft CPC status in separate multivariable models; HCT-CI, prior response, and prior MRD response were included in this model.

**Table 3:** Summary of multivariable assessments for overall survival (OS).

Parameter	Hazard ratio (95% CI)	*p* value
Autograft CPC status		
Positive vs. Negative	1.84 (1.24, 2.74)	0.003
ISS		
II vs. I	1.53 (0.94, 2.49)	0.09
III vs. I	2.22 (1.36, 3.61)	0.12
KPS		
≥90 vs. <90	0.85 (0.59, 1.22)	0.39
HCT-CI		
>3 vs. ≤3	1.50 (1.01, 2.22)	0.044
Prior response		
PR or worse vs. VGPR or better	1.24 (0.86, 1.80)	0.25
Prior MRD response		
Positive vs. Negative	1.75 (1.11, 2.77)	0.016
Maintenance therapy^a^		
Yes vs. No	0.68 (0.46, 1.00)	0.050
Positive bag infused^a^		
Yes vs. No	1.47 (0.86, 2.49)	0.16
Degree of autograft MRD positivity – average^a^	1.37 (1.13, 1.67)	0.001

*CI* Confidence interval, *MRD* Minimal residual disease, *ISS* international staging system, *KPS* Karnofsky performance status, *HCT-CI* Hematopoietic cell transplant comorbidity index.

^a^Included in the model as a time-dependent covariate.

^b^Replaces autograft CPC status in separate multivariable models; Prior response and prior MRD response were included in this model.

Use of chemotherapy for stem cell mobilization did not impact the rate of CPC- in the collected autografts (83% with and 78% without chemotherapy; *p* = 0.39). Eighty-nine percent (*n* = 141) of patients who received VRD induction had CPC- autografts, whereas 78% of patients who received VCD and 83% of those who received KRD had CPC- autografts (Table 1). Use of VRD for induction was not associated with improved PFS (HR 1.14 [0.88–1.47]; *p* = 0.32) or OS (HR 1.08 [0.77–1.51]; *p* = 0.67) compared to other induction regimens. Within the subgroup of patients who received VRD induction, autograft CPC status was highly predictive of both PFS (HR 3.10 [1.75–5.49]; *p* < 0.001) and OS (HR 2.66 [1.33–5.33]; *p* = 0.006).

Since a different method of CPC detection was used between 2008–2011 (*n* = 55) and 2012–2018 (*n* = 361), we repeated the analyses while excluding patients who were transplanted in the earlier era and obtained similar results. When considering only patients transplanted between 2012 and 2018, the CPC + group had inferior median PFS (15.4 vs. 35.5 months, *p* < 0.001) and median OS (39.3 months vs. OS not reached, *p* < 0.001) compared to the CPC− group.

## Discussion

To the best of our knowledge, this is the largest study to date to evaluate the impact of autograft contamination by CPC in patients with MM undergoing autoHCT. It is also the first to focus only on patients with HRMM. We found that in patients with HRMM undergoing autoHCT, presence of CPC in the autograft was associated with inferior PFS and OS. Furthermore, the degree of autograft CPC positivity was associated with worse outcomes. Through MVA, we found a significant association between autograft CPC positivity and patient outcomes, which was also observed in patients who had already achieved ≥VGPR or MRD negative ≥VGPR prior to autoHCT.

Most patients with MM eventually relapse after transplant, especially those with high-risk cytogenetic abnormalities. Infusion of CPC in the autograft is considered a potential source of relapse. Several studies, including meta-analyses, have shown that MRD detection in the bone marrow by NGF or next-generation sequencing (NGS), performed at various time points in both transplant-eligible and ineligible patients, predicts worse outcomes [9–12]. In addition, separate reports by the EBMT [13] and the CIBMTR [14] showed lower relapse rates after syngeneic hematopoietic stem cell transplants compared to autologous transplants. This could partly be due to the absence of CPC contamination in syngeneic grafts.

In the present study, patients in the CPC+ group were more likely to have del17p and less likely to have achieved a VGPR after induction. However, when the analysis was restricted to CPC + patients who had achieved ≥VGPR or MRD-negative ≥VGPR after induction, presence of CPC in the autograft still predicted worse outcomes. Compared to MRD detection in the bone marrow, detection of CPC in the autograft is less invasive and is not limited by focal plasma cell infiltration [15]. Yet, for the most part, hematological responses prior to autoHCT and at day 100 after autoHCT within the CPC+ and CPC− groups retained their predictive impact for both PFS and OS. This is in contrast to an analysis of the PETHEMA/GEM2012MENOS65 study by Jimenez-Ubieto et al. that showed that patients with bone marrow MRD positivity had similarly poor survival outcomes, irrespective of their hematological response [16].

Bal et al. reported a trend toward higher rates of autograft CPC negativity in patients who received KRD induction, compared to VRD (81% vs. 57%, respectively; *p* = 0.25) [17]. In contrast, we found a higher rate of autograft CPC negativity in patients who received VRD. Differences between the study cohorts may account for these conflicting results, such as our study including only patients with high-risk cytogenetics, while only 16% of the patients in the analysis by Bal et al. had high-risk cytogenetics. There was no significant difference in the median duration of induction treatment between the CPC+ and CPC− groups, thereby ruling out the role of longer induction in the CPC− group.

Our study raises the question of whether purging of CPC could mitigate the adverse impact of CPC in the autografts. Several preclinical studies have suggested potential efficacy of various ex vivo purging methods [18–20]. However, clinical experience with purging in MM has had disappointing results, with several trials failing to show an advantage of ex-vivo purging [5, 21]. A phase III trial published in 2001 demonstrated that CD34 selection using a Ceprate-R Stem Cell concentrator device resulted in a 3-log reduction in MM cells in the autografts, though this did not translate into improved PFS or OS [22, 23]. Of note, 40 of the 111 enrolled patients in that study could not be assessed for autograft contamination due to various technical issues. With the availability of newer and more reliable technologies, ex-vivo purging of CPC could be revisited.

Administering chemotherapy prior to autograft collection could have an in-vivo purging effect, which may reduce CPC in the graft. However, a study comparing cyclophosphamide-based chemo-mobilization to growth factor-only mobilization showed increased toxicity with chemo-mobilization without improving EFS or OS [24]. A phase I trial examining the role of in-vivo purging by adding bortezomib to granulocyte colony-stimulating factor (G-CSF) failed to show any benefit [25]. In our study, we did not observe a difference in the rate of CPC positivity between chemo-mobilization or growth factor-only mobilization.

Our study has several limitations. First, being a retrospective analysis, it has inherent issues with patient and treatment heterogeneity, missing data, and patient selection bias. Second, since we focused on patients with HRMM, the findings may not be applicable to patients with standard risk MM. Furthermore, the detection of CPC in the autografts was limited by the sensitivity of the 6 color flow cytometry technique in most of our cohort. It is possible that a more sensitive method could identify an even smaller group of CPC− patients, with deeper response to induction and even better survival outcomes.

In conclusion, the current study shows a major impact of CPC in the autograft on post-autoHCT outcomes in HRMM. Both the presence and degree of CPC in the autograft were highly predictive of inferior PFS and OS, including in those who had achieved ≥VGPR and MRD-negative CR or VGPR prior to autoHCT. Novel strategies for purging of CPC could improve patient outcomes.

## Supplementary information

Supplemental Table 1

Supplemental Table 2

Supplemental Figures

## Data Availability

The datasets generated during and/or analyzed during the current study are available from the corresponding author on reasonable request.

## References

[1] Callander NS, Baljevic M, Adekola K, Anderson LD, Campagnaro E, Castillo JJ (2022). NCCN Guidelines(R) Insights: Multiple Myeloma, Version 3.2022. J Natl Compr Canc Netw.

[2] Scott EC, Hari P, Sharma M, Le-Rademacher J, Huang J, Vogl D (2016). Post-Transplant Outcomes in High-Risk Compared with Non-High-Risk Multiple Myeloma: A CIBMTR Analysis. Biol Blood Marrow Transpl.

[3] Vogel W, Kopp HG, Kanz L, Einsele H (2005). Myeloma cell contamination of peripheral blood stem-cell grafts can predict the outcome in multiple myeloma patients after high-dose chemotherapy and autologous stem-cell transplantation. J Cancer Res Clin Oncol.

[4] Kopp HG, Yildirim S, Weisel KC, Kanz L, Vogel W (2009). Contamination of autologous peripheral blood progenitor cell grafts predicts overall survival after high-dose chemotherapy in multiple myeloma. J Cancer Res Clin Oncol.

[5] Galimberti S, Morabito F, Guerrini F, Palumbo GA, Azzara A, Martino M (2003). Peripheral blood stem cell contamination evaluated by a highly sensitive molecular method fails to predict outcome of autotransplanted multiple myeloma patients. Br J Haematol.

[6] Gertz MA, Witzig TE, Pineda AA, Greipp PR, Kyle RA, Litzow MR (1997). Monoclonal plasma cells in the blood stem cell harvest from patients with multiple myeloma are associated with shortened relapse-free survival after transplantation. Bone Marrow Transpl.

[7] Wuilleme S, Lok A, Robillard N, Dupuis P, Stocco V, Migne H (2016). Assessment of tumoral plasma cells in apheresis products for autologous stem cell transplantation in multiple myeloma. Bone Marrow Transpl.

[8] Kostopoulos IV, Eleutherakis-Papaiakovou E, Rousakis P, Ntanasis-Stathopoulos I, Panteli C, Orologas-Stavrou N (2021). Aberrant Plasma Cell Contamination of Peripheral Blood Stem Cell Autografts, Assessed by Next-Generation Flow Cytometry, Is a Negative Predictor for Deep Response Post Autologous Transplantation in Multiple Myeloma; A Prospective Study in 199 Patients. Cancers (Basel).

[9] Flores-Montero J, Sanoja-Flores L, Paiva B, Puig N, Garcia-Sanchez O, Bottcher S (2017). Next Generation Flow for highly sensitive and standardized detection of minimal residual disease in multiple myeloma. Leukemia.

[10] Korthals M, Sehnke N, Kronenwett R, Bruns I, Mau J, Zohren F (2012). The level of minimal residual disease in the bone marrow of patients with multiple myeloma before high-dose therapy and autologous blood stem cell transplantation is an independent predictive parameter. Biol Blood Marrow Transpl.

[11] Munshi NC, Avet-Loiseau H, Anderson KC, Neri P, Paiva B, Samur M (2020). A large meta-analysis establishes the role of MRD negativity in long-term survival outcomes in patients with multiple myeloma. Blood Adv.

[12] Munshi NC, Avet-Loiseau H, Rawstron AC, Owen RG, Child JA, Thakurta A (2017). Association of Minimal Residual Disease With Superior Survival Outcomes in Patients With Multiple Myeloma: A Meta-analysis. JAMA Oncol.

[13] Gahrton G, Svensson H, Bjorkstrand B, Apperley J, Carlson K, Cavo M (1999). Syngeneic transplantation in multiple myeloma - a case-matched comparison with autologous and allogeneic transplantation. European Group for Blood and Marrow Transplantation. Bone Marrow Transpl.

[14] Bashey A, Perez WS, Zhang MJ, Anderson KC, Ballen K, Berenson JR (2008). Comparison of twin and autologous transplants for multiple myeloma. Biol Blood Marrow Transpl.

[15] Cengiz Seval G, Beksac M (2022). Is Quantification of Measurable Clonal Plasma Cells in Stem Cell Grafts (gMRD) Clinically Meaningful?. Front Oncol.

[16] Jimenez-Ubieto A, Paiva B, Puig N, Cedena MT, Martinez-Lopez J, Oriol A (2021). Validation of the International Myeloma Working Group standard response criteria in the PETHEMA/GEM2012MENOS65 study: are these times of change?. Blood.

[17] Bal S, Landau HJ, Shah GL, Scordo M, Dahi P, Lahoud OB (2020). Stem Cell Mobilization and Autograft Minimal Residual Disease Negativity with Novel Induction Regimens in Multiple Myeloma. Biol Blood Marrow Transpl.

[18] Bartee E, Chan WM, Moreb JS, Cogle CR, McFadden G (2012). Selective purging of human multiple myeloma cells from autologous stem cell transplantation grafts using oncolytic myxoma virus. Biol Blood Marrow Transpl.

[19] Lee AJ, Kim SG (2019). Selective purging of human multiple myeloma cells from peripheral blood mononuclear cells: a preliminary study. J Blood Med.

[20] Yang H, Robinson SN, Nieto Y, Jones RJ, Gocke CD, Lu J (2011). Ex vivo graft purging and expansion of autologous blood progenitor cell products from patients with multiple myeloma. Cancer Res.

[21] Gupta D, Bybee A, Cooke F, Giles C, Davis JG, McDonald C (1999). CD34+-selected peripheral blood progenitor cell transplantation in patients with multiple myeloma: tumour cell contamination and outcome. Br J Haematol.

[22] Bourhis JH, Bouko Y, Koscielny S, Bakkus M, Greinix H, Derigs G (2007). Relapse risk after autologous transplantation in patients with newly diagnosed myeloma is not related with infused tumor cell load and the outcome is not improved by CD34+ cell selection: long term follow-up of an EBMT phase III randomized study. Haematologica.

[23] Stewart AK, Vescio R, Schiller G, Ballester O, Noga S, Rugo H (2001). Purging of autologous peripheral-blood stem cells using CD34 selection does not improve overall or progression-free survival after high-dose chemotherapy for multiple myeloma: results of a multicenter randomized controlled trial. J Clin Oncol.

[24] Tuchman SA, Bacon WA, Huang LW, Long G, Rizzieri D, Horwitz M (2015). Cyclophosphamide-based hematopoietic stem cell mobilization before autologous stem cell transplantation in newly diagnosed multiple myeloma. J Clin Apher.

[25] Ghobadi A, Fiala MA, Rettig M, Schroeder M, Uy GL, Stockerl-Goldstein K (2019). A Phase I Study of the Safety and Feasibility of Bortezomib in Combination With G-CSF for Stem Cell Mobilization in Patients With Multiple Myeloma. Clin Lymphoma Myeloma Leuk.

