# Supplementary material for: Impact of clonal plasma cells in autografts on outcomes in high-risk multiple myeloma patients
Source: Blood Cancer J. 2023 May 3;13(1):68. doi: 10.1038/s41408-023-00842-6 (PMC10156676; doi:10.1038/s41408-023-00842-6)
Supplement: Supplementary file 1 — Supplemental Table 1 [file 41408_2023_842_MOESM1_ESM.docx]

Supplementary Table 1. Summary of progression free survival: univariate assessments.

| **Parameter** | **Median (95% CI)** | **p-value** | **Hazard Ratio (95% CI)** | **p-value** |
| --- | --- | --- | --- | --- |
|  | **(in months)** |  |  |  |
| **PFS-All** | 26.3 (23.7, 31.9) |  |  |  |
| **Autograft CPC status** |  | < 0.001 |  |  |
| Negative | 32.1 (26.6, 39.1) |  | *ref* |  |
| Positive | 12.8 (10.3, 18.0) |  | 2.52 (1.89, 3.37) | < 0.001 |
| **Positive bag infused** |  | < 0.001 |  |  |
| No | 31.2 (25.2, 33.8) |  | *ref* |  |
| Yes | 12.8 (8.9, 17.3) |  | 3.01 (2.08, 4.37) | < 0.001 |
| **Degree of autograft CPC positivity - average** |  |  | 1.50 (1.18, 1.90) | < 0.001 |
| **Degree of autograft CPC positivity - maximum** |  |  | 1.50 (1.19, 1.91) | < 0.001 |
| **Gender** |  | 0.14 |  |  |
| Male | 30.3 (24.8, 35.9) |  | *ref* |  |
| Female | 22.0 (18.2, 30.7) |  | 1.20 (0.94, 1.52) | 0.14 |
| **Age at autoHCT** |  |  | 1.00 (0.99, 1.02) | 0.64 |
| **ISS** |  | 0.032 |  |  |
| I | 33.8 (28.3, 48.1) |  | *ref* |  |
| II | 24.5 (18.3, 31.6) |  | 1.43 (1.04, 1.97) | 0.026 |
| III | 18.7 (12.8, 28.7) |  | 1.49 (1.06, 2.08) | 0.020 |
| **Induction treatment** |  | 0.32 |  |  |
| VRD | 28.3 (23.7, 39.6) |  | *ref* |  |
| Other | 26.3 (20.2, 32.8) |  | 1.14 (0.88, 1.47) | 0.32 |
| **KPS** |  | 0.06 |  |  |
| < 90 | 22.9 (17.9, 31.2) |  | *ref* |  |
| ≥ 90 | 28.4 (24.5, 37.2) |  | 0.79 (0.61, 1.02) | 0.07 |
| **HCT-CI** |  | 0.08 |  |  |
| ≤ 3 | 28.7 (24.1, 33.6) |  | *ref* |  |
| > 3 | 22.8 (16.5, 31.9) |  | 1.28 (0.97, 1.69) | 0.08 |
| **Prior response** |  | < 0.001 |  |  |
| CR | 41.2 (25.7, 65.8) |  | *ref* |  |
| VGPR | 31.9 (25.0, 40.9) |  | 1.33 (0.86, 2.05) | 0.21 |
| PR | 24.8 (19.0, 31.6) |  | 1.76 (1.13, 2.73) | 0.012 |
| SD | 32.8 (9.6, 70.7) |  | 1.58 (0.76, 3.30) | 0.22 |
| PD | 7.0 (3.7, 9.2) |  | 7.31 (4.31, 12.40) | < 0.001 |
| **Prior MRD response** |  | < 0.001 |  |  |
| Negative | 42.3 (32.1, 71.9) |  | *ref* |  |
| Positive | 21.3 (18.4, 25.0) |  | 1.98 (1.50, 2.62) | < 0.001 |
| **Prior response ≥ VGPR** |  | < 0.001 |  |  |
| CPC Negative | 39.1 (31.9, 48.2) |  | *ref* |  |
| CPC Positive | 12.8 (8.9, 21.6) |  | 3.38 (2.05, 5.58) | < 0.001 |
| **Prior response < VGPR** |  | 0.001 |  |  |
| CPC Negative | 24.5 (18.3, 32.7) |  | *ref* |  |
| CPC Positive | 12.8 (8.2, 18.0) |  | 1.80 (1.25, 2.60) | 0.002 |
| **Prior MRD negative ≥ VGPR** |  | 0.003 |  |  |
| CPC Negative | 71.9 (39.1, NE) |  | *ref* |  |
| CPC Positive | 11.6 (3.1, 52.2) |  | 4.21 (1.50, 11.81) | 0.006 |
| **Prior MRD/response – other** |  | < 0.001 |  |  |
| CPC Negative | 26.5 (22.0, 32.0) |  | *ref* |  |
| CPC Positive | 12.9 (9.6, 18.1) |  | 2.09 (1.53, 2.86) | < 0.001 |
| **Induction treatment – VRD** |  | < 0.001 |  |  |
| CPC Negative | 31.9 (25.7, 47.7) |  | *ref* |  |
| CPC Positive | 15.4 (8.7, 19.4) |  | 3.10 (1.75, 5.49) | < 0.001 |
| **Induction treatment – Other** |  | < 0.001 |  |  |
| CPC Negative | 33.8 (26.3, 41.1) |  | *ref* |  |
| CPC Positive | 12.0 (8.9, 18.0) |  | 2.35 (1.65, 3.34) | < 0.001 |
| **Maintenance therapy^a^** |  |  |  |  |
| Yes vs. No | - | - | 1.14 (0.85, 1.53) | 0.37 |

**Abbreviations:** PFS=Progression free survival, *ref*=reference group, CI=Confidence interval, MRD=Minimal residual disease, AutoHCT=Autologous hematopoietic stem cell transplant, ISS=international staging system, KPS=Karnofsky performance status, HCT-CI=Hematopoietic cell transplant comorbidity index, CR=Complete response, VGPR=Very good partial response, PR=Partial response, SD=Stable disease, PD=Progression of disease, NE=not estimated/not reached, VRD=Bortezomib/lenalidomide and dexamethasone.

^a^Included in the model as a time-dependent covariate.
