# Supplementary material for: Impact of clonal plasma cells in autografts on outcomes in high-risk multiple myeloma patients
Source: Blood Cancer J. 2023 May 3;13(1):68. doi: 10.1038/s41408-023-00842-6 (PMC10156676; doi:10.1038/s41408-023-00842-6)
Supplement: Supplementary file 2 — Supplemental Table 2 [file 41408_2023_842_MOESM2_ESM.docx]

Supplementary Table 2. Summary of overall survival: univariate Assessments.

| **Parameter** | **Median OS (95% CI)** | **p-value** | **Hazard Ratio (95% CI)** | **p-value** |
| --- | --- | --- | --- | --- |
|  | **(in months)** |  |  |  |
| **OS-All** | 71.9 (65.5, 85.9) |  |  |  |
| **Autograft CPC status** |  | <0.001 |  |  |
| Negative | 81.2 (69.7, NE) |  | *ref* |  |
| Positive | 36.4 (28.0, 51.4) |  | 2.31 (1.61, 3.31) | < 0.001 |
| **Positive bag infused** |  | 0.009 |  |  |
| No | 73.7 (66.6, 92.4) |  | *ref* |  |
| Yes | 30.9 (27.4, 52.0) |  | 1.90 (1.17, 3.09) | 0.010 |
| **Degree of autograft CPC positivity - average** |  |  | 1.38 (1.14, 1.67) | < 0.001 |
| **Degree of autograft CPC positivity - maximum** |  |  | 1.39 (1.15, 1.68) | < 0.001 |
| **Gender** |  | 0.37 |  |  |
| Male | 70.2 (63.2, 85.9) |  | *ref* |  |
| Female | 71.9 (44.9, NE) |  | 1.16 (0.84, 1.60) | 0.37 |
| **Age at autoHCT** |  |  | 1.01 (0.99, 1.03) | 0.31 |
| **ISS** |  | 0.004 |  |  |
| I | 73.7 (70.2, NE) |  | *ref* |  |
| II | 76.6 (52.0, 103.6) |  | 1.45 (0.92, 2.29) | 0.11 |
| III | 67.7 (38.7, NE) |  | 2.12 (1.34, 3.34) | 0.001 |
| **Induction treatment** |  | 0.67 |  |  |
| VRD | 81.2 (51.2, NE) |  | *ref* |  |
| Other | 70.2 (63.2, 85.9) |  | 1.08 (0.77, 1.51) | 0.67 |
| **KPS** |  | 0.010 |  |  |
| < 90 | 63.2 (52.0, 71.9) |  | *ref* |  |
| ≥ 90 | 103.6 (69.7, NE) |  | 0.65 (0.46, 0.90) | 0.011 |
| **HCT-CI** |  | 0.004 |  |  |
| ≤ 3 | 76.6 (67.7, NE) |  | *ref* |  |
| > 3 | 52.0 (39.3, 72.2) |  | 1.67 (1.17, 2.38) | 0.005 |
| **Prior response** |  | <0.001 |  |  |
| CR | 71.5 (51.1, NE) |  | *ref* |  |
| VGPR | 92.4 (69.7, NE) |  | 1.15 (0.63, 2.11) | 0.65 |
| PR | 76.6 (60.4, 103.6) |  | 1.42 (0.77, 2.61) | 0.26 |
| SD | 72.2 (19.6, NE) |  | 1.59 (0.60, 4.20) | 0.35 |
| PD | 19.4 (13.5, 38.7) |  | 4.74 (2.45, 9.16) | < 0.001 |
| **Prior MRD response** |  | <0.001 |  |  |
| Negative | 92.4 (73.7, NE) |  | *ref* |  |
| Positive | 63.2 (51.2, 72.2) |  | 1.99 (1.34, 2.96) | < 0.001 |
| **Prior response ≥ VGPR** |  | 0.010 |  |  |
| CPC Negative | 92.4 (71.5, NE) |  | *ref* |  |
| CPC Positive | 36.4 (28.0, 71.9) |  | 2.29 (1.20, 4.40) | 0.013 |
| **Prior response < VGPR** |  | 0.004 |  |  |
| CPC Negative | 70.2 (57.1, 103.6) |  | *ref* |  |
| CPC Positive | 35.7 (23.6, 44.9) |  | 1.92 (1.22, 3.00) | 0.004 |
| **Prior MRD negative ≥ VGPR** |  | < 0.001 |  |  |
| CPC Negative | 92.4 (73.7, NE) |  | *ref* |  |
| CPC Positive | 19.5 (6.7, 67.7) |  | 7.04 (2.05, 24.20) | 0.002 |
| **Prior MRD/response – other** |  | < 0.001 |  |  |
| CPC Negative | 70.2 (57.1, 103.6) |  | *ref* |  |
| CPC Positive | 36.4 (28.7, 51.4) |  | 1.94 (1.31, 2.89) | 0.001 |
| **Induction treatment – VRD** |  | 0.004 |  |  |
| CPC Negative | 81.2 (55.1, NE) |  | *ref* |  |
| CPC Positive | 29.3 (19.6, 40.6) |  | 2.66 (1.33, 5.33) | 0.006 |
| **Induction treatment – Other** |  | < 0.001 |  |  |
| CPC Negative | 73.7 (69.7, NE) |  | *ref* |  |
| CPC Positive | 37.8 (28.0, 63.2) |  | 2.21 (1.42, 3.44) | < 0.001 |
| **Maintenance therapy^a^** |  |  |  |  |
| Yes vs. No | - | - | 0.60 (0.43, 0.85) | 0.004 |

**Abbreviations:** OS=Overall survival, CI=Confidence interval, NE=not estimated/not reached, *ref*=reference group, MRD=Minimal residual disease, AutoHCT=Autologous hematopoietic stem cell transplant, ISS=international staging system, KPS=Karnofsky performance status, HCT-CI=Hematopoietic cell transplant comorbidity index, CR=Complete response, VGPR=Very good partial response, PR=Partial response, SD=Stable disease, PD=Progression of disease, VRD=Bortezomib/lenalidomide and dexamethasone.

^a^ Included in the model as a time-dependent covariate.
