# Supplementary material for: Impact of clonal plasma cells in autografts on outcomes in high-risk multiple myeloma patients
Source: Blood Cancer J. 2023 May 3;13(1):68. doi: 10.1038/s41408-023-00842-6 (PMC10156676; doi:10.1038/s41408-023-00842-6)

**Supplementary Figure 1. Progression-free survival, by hematological response prior to autoHCT;** **CPC- Patients**


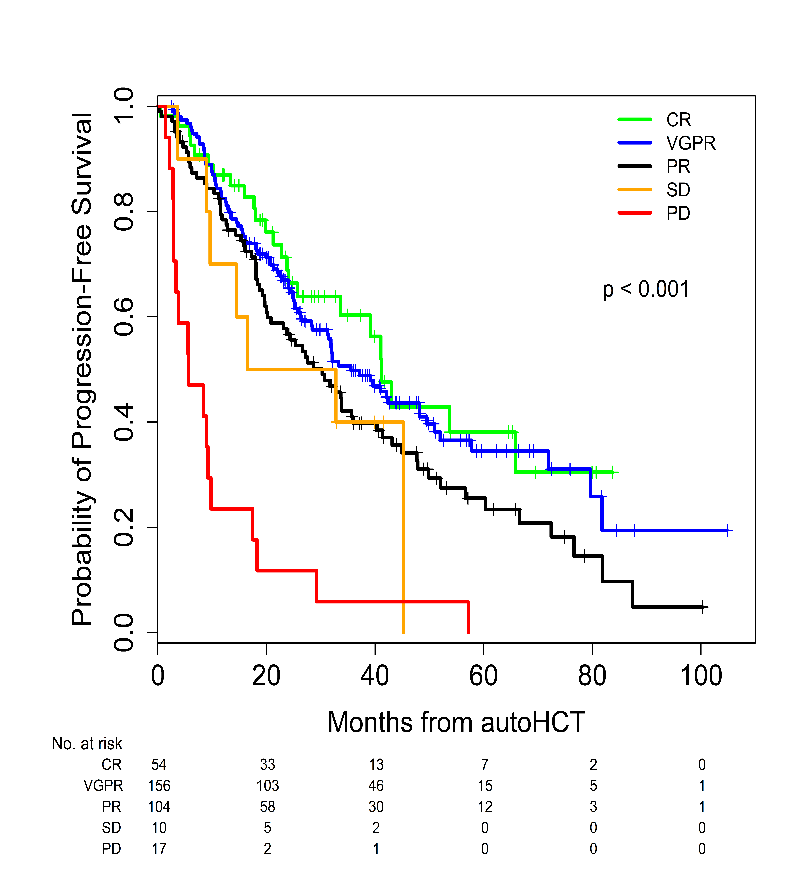


**Supplementary Figure 2. Progression-free survival, by hematological response prior to autoHCT; CPC+ Patients**


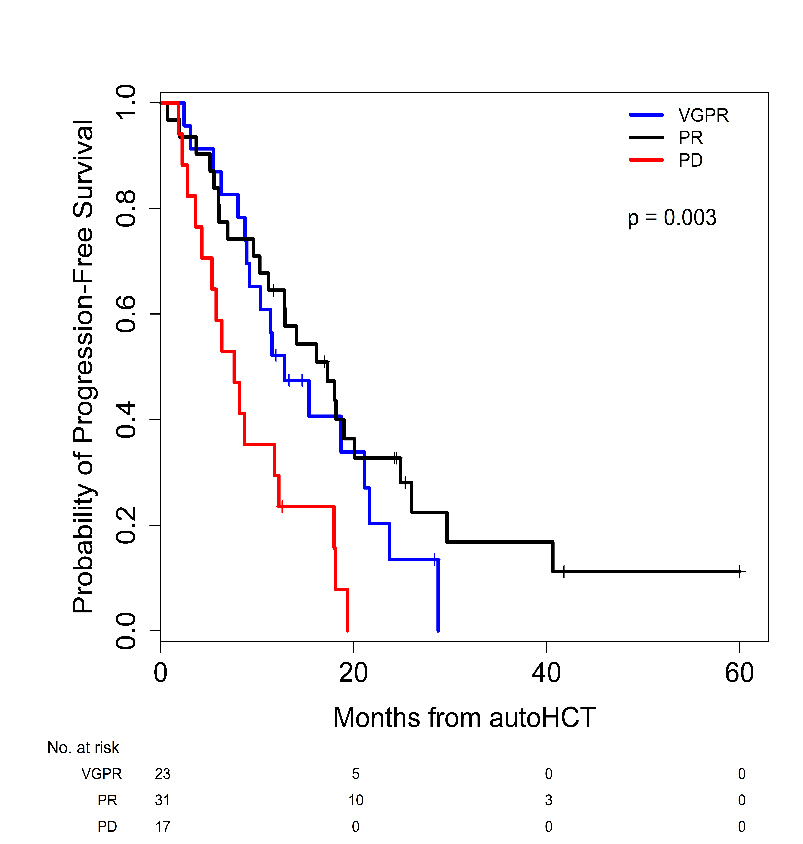


**Supplementary Figure 3. Progression-free survival, by hematological response at day 100 after autoHCT; CPC- Patients**


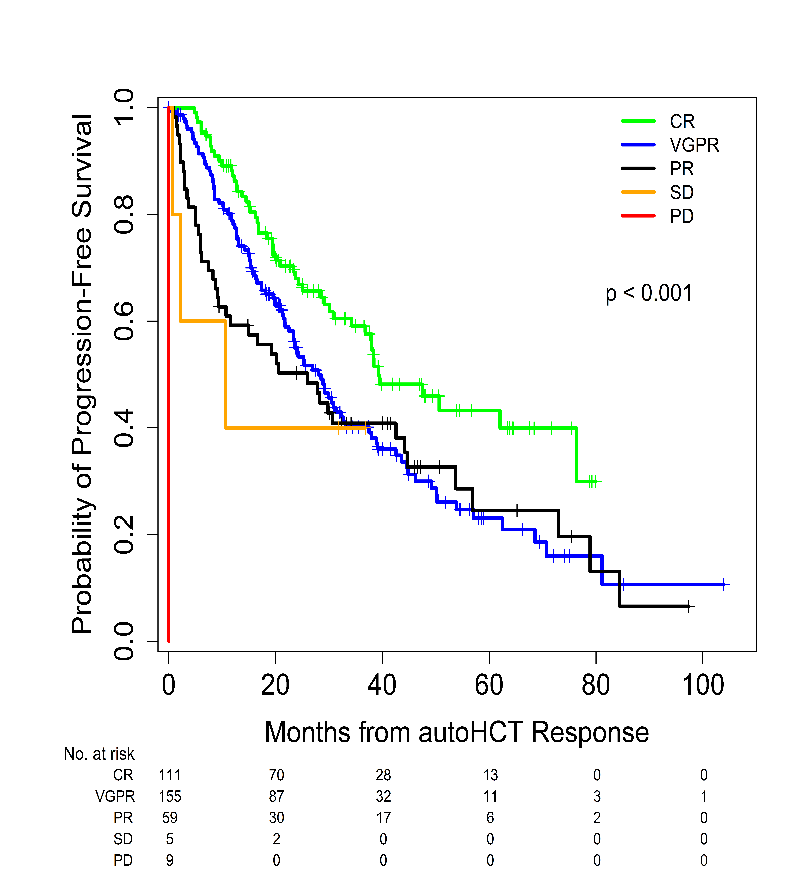


**Supplementary Figure 4. Progression-free survival, by hematological response at day 100 after autoHCT; CPC+ Patients**


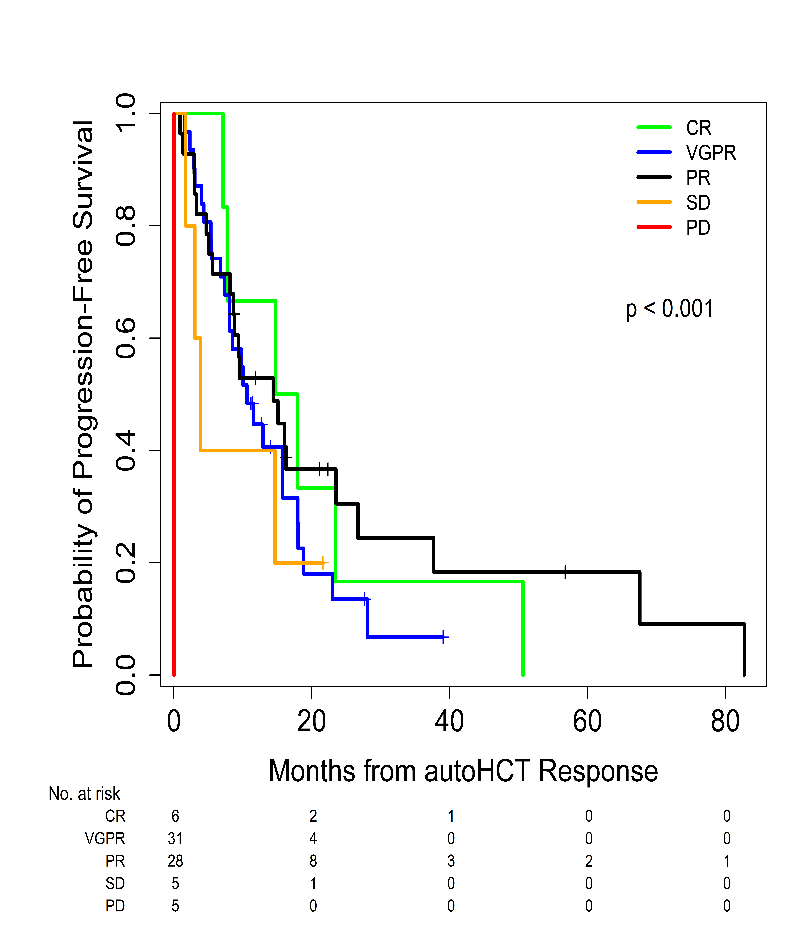


**Supplementary Figure 5. Overall survival, by hematological response prior to autoHCT; CPC- Patients**


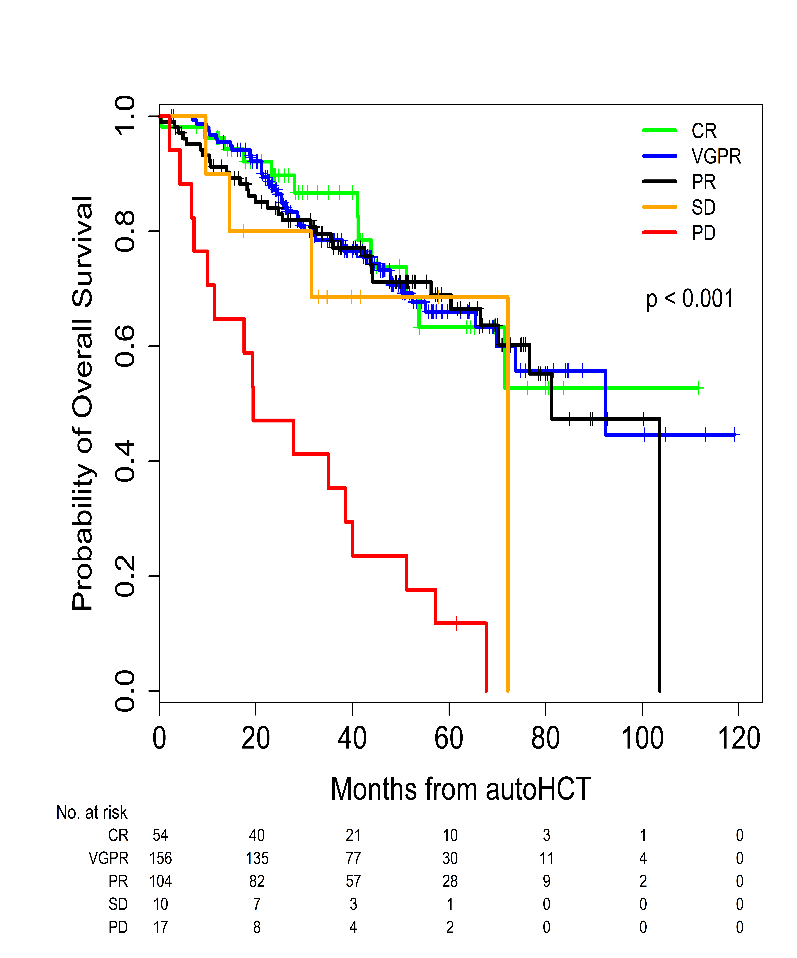


**Supplementary Figure 6. Overall survival, by hematological response prior to autoHCT; CPC+ Patients**


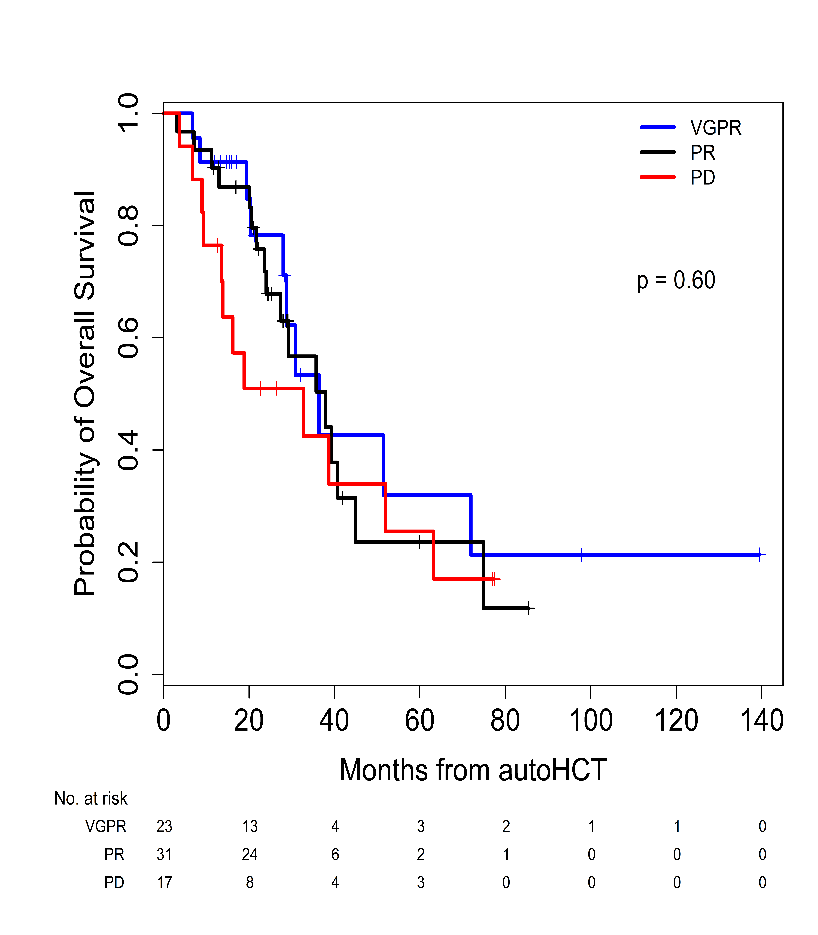


**Supplementary Figure 7. Overall survival, by hematological response at day 100 after autoHCT; CPC- patients.**


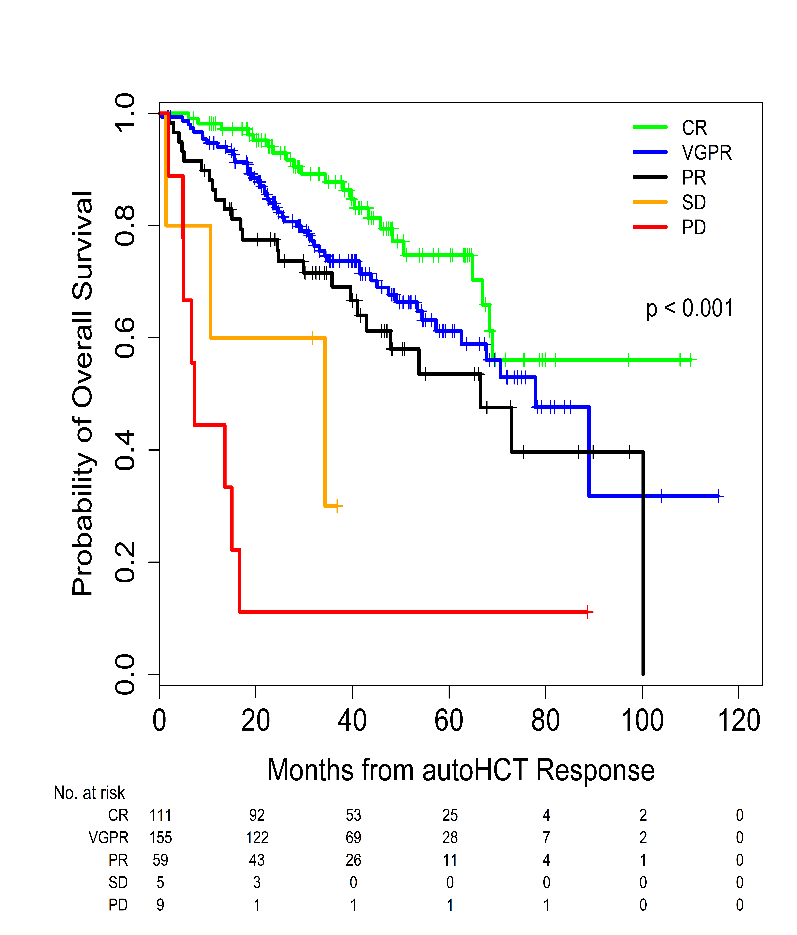


**Supplementary Figure 8. Overall survival, by hematological response at day 100 after autoHCT; CPC+ patients.**


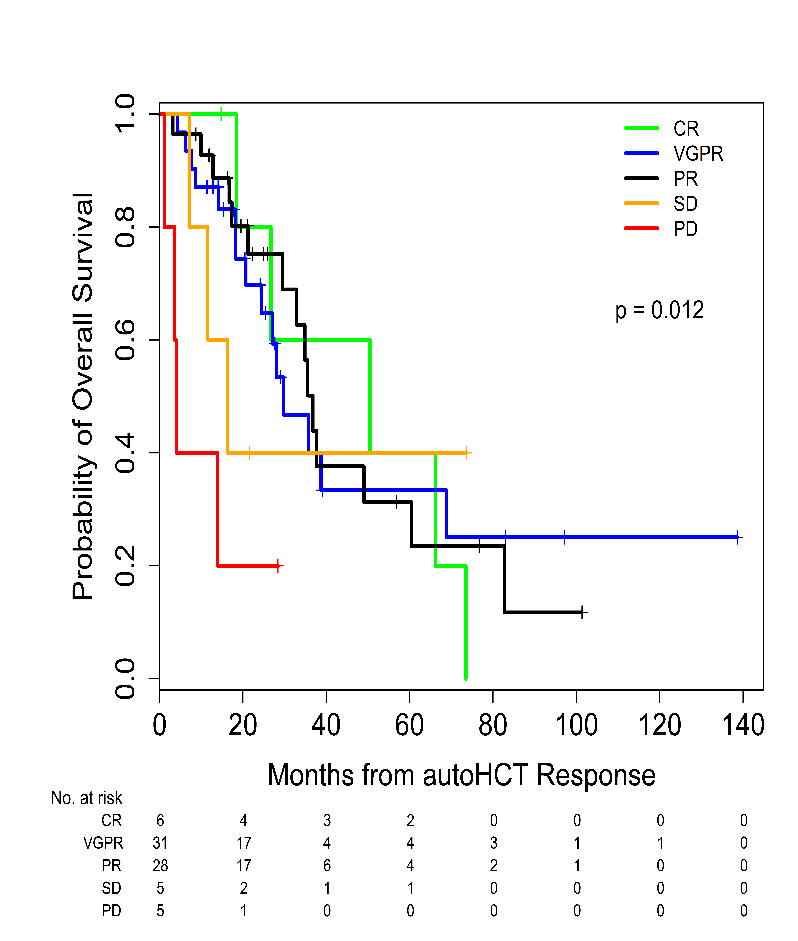

Supplement: Supplementary file 3 — Supplemental Figures [file 41408_2023_842_MOESM3_ESM.docx]
